# Correction: Lineage tracing of human B cells reveals the in vivo landscape of human antibody class switching

**DOI:** 10.7554/eLife.23066

**Published:** 2016-11-09

**Authors:** Felix Horns, Christopher Vollmers, Derek Croote, Sally F Mackey, Gary E Swan, Cornelia L Dekker, Mark M Davis, Stephen R Quake

Horns F, Vollmers C, Croote D, Mackey SF, Swan GE, Dekker CL, Davis MM, Quake SR. 2016. Lineage tracing of human B cells reveals the in vivo landscape of human antibody class switching. *eLife* **5**:e16578. doi: 10.7554/eLife.16578.

Published 2, August 2016

In the originally published article, the primer sequences used for PCR amplification of immunoglobulin heavy chain genes (IGH) which bind in the constant region were reported incorrectly in Table 5. The correct oligonucleotide sequences are now reported in the corrected Table 5.

In the 'Library preparation' section of 'Materials and methods', the text “a pooled set of five isotype-specific IGH constant region primers that contain 8 random nts (Table 5)” has been changed to “a pooled set of ten isotype-specific IGH constant region primers that contain 8 or 12 random nts (Table 5)”.

The article has been corrected accordingly.

